# Lifetime prevalence, associated factors, and circumstances of non-volitional sex in women and men in Britain: findings from the third National Survey of Sexual Attitudes and Lifestyles (Natsal-3)

**DOI:** 10.1016/S0140-6736(13)62300-4

**Published:** 2013-11-30

**Authors:** Wendy Macdowall, Lorna J Gibson, Clare Tanton, Catherine H Mercer, Ruth Lewis, Soazig Clifton, Nigel Field, Jessica Datta, Kirstin R Mitchell, Pam Sonnenberg, Bob Erens, Andrew J Copas, Andrew Phelps, Philip Prah, Anne M Johnson, Kaye Wellings

**Affiliations:** a Centre for Sexual and Reproductive Health Research, Department of Social and Environmental Health Research, London School of Hygiene and Tropical Medicine, London, UK; b Department of Health Services Research and Policy, London School of Hygiene and Tropical Medicine, London, UK; c Research Department of Infection and Population Health, University College London, London, UK; d NatCen Social Research, London, UK

## Abstract

**Background:**

Sexual violence is increasingly recognised as a public health issue. Information about prevalence, associated factors, and consequences for health in the population of Britain (England, Scotland, and Wales) is scarce. The third National Survey of Sexual Health Attitudes and Lifestyles (Natsal-3) is the first of the Natsal surveys to include questions about sexual violence and the first population-based survey in Britain to explore the issue outside the context of crime.

**Methods:**

Between Sept 6, 2010, and Aug 31, 2012, we did a probability sample survey of women and men aged 16–74 years living in Britain. We asked participants about their experience of sex against their will since age 13 years and the circumstances surrounding the most recent occurrence. We explored associations between ever experiencing non-volitional sex and a range of sociodemographic, health, and behavioural factors. We used logistic regression to estimate age-adjusted odds ratios to analyse factors associated with the occurrence of completed non-volitional sex in women and men.

**Findings:**

We interviewed 15 162 people. Completed non-volitional sex was reported by 9·8% (95% CI 9·0–10·5) of women and 1·4% (1·1–1·7) of men. Median age (interdecile range) at most recent occurrence was 18 years (14–32) for women and 16 years (13–30) for men. Completed non-volitional sex varied by family structure and, in women, by age, education, and area-level deprivation. It was associated with poor health, longstanding illness or disability, and treatment for mental health conditions, smoking, and use of non-prescription drugs in the past year in both sexes, and with binge drinking in women. Completed non-volitional sex was also associated with reporting of first heterosexual intercourse before 16 years of age, same-sex experience, more lifetime sexual partners, ever being diagnosed with a sexually transmitted infection, and low sexual function in both sexes, and, in women, with abortion and pregnancy outcome before 18 years of age. In most cases, the person responsible was known to the individual, although the nature of the relationship differed by age at most recent occurrence. Participants who were younger at interview were more likely to have told someone about the event and to have reported it to the police than were older participants.

**Interpretation:**

These data provide the first population prevalence estimates of non-volitional sex in Britain. We showed it to be mainly an experience of young age and strongly associated with a range of adverse health outcomes in both women and men.

**Funding:**

Grants from the UK Medical Research Council and the Wellcome Trust, with support from the Economic and Social Research Council and the Department of Health.

## Introduction

Sexual violence is a violation of fundamental human rights, and recognition of the global magnitude of the problem has grown during the past two decades.^1^ It encompasses a range of acts, from verbal harassment to forced penetration, and different degrees of coercion, from intimidation to physical force.^2^ It can be experienced by people of all ages as a single event or as part of a pattern of victimisation lasting for months or years. The potential health effects are similarly broad ranging, and include physical, sexual and reproductive, and mental health sequelae.^1^, ^3^, ^4^, ^5^ As the human, economic, and wider social costs are becoming better understood,^1^, ^2^, ^3^, ^4^, ^5^ sexual violence is increasingly recognised as a global public health issue that needs urgent attention.^1^, ^6^

So far, most research has focused on the experience of women and on sexual violence within the contexts of so-called date rape^7^ and of intimate partner violence (IPV),^1^, ^3^, ^4^ which also includes physical and emotional violence and controlling behaviour.^2^ Less is known about other forms of sexual violence or about sexual violence in isolation from other forms of abuse within IPV.^1^, ^4^ Less still is known about men as victims.^8^

Measurement of the prevalence of sexual violence—rape in particular—and by extension its consequences for health, is challenging for many reasons;^9^ sexual violence is highly stigmatised and is among the few crimes in which the victim might also be blamed.^9^ Furthermore, people who have been victims of what is legally defined as rape might not acknowledge it as such.^10^ General agreement exists that the use of the term rape should be avoided in research because it is highly subjective and likely to lead to under-reporting; neutral and behaviourally specific terms are preferred.^9^, ^11^

The National Surveys of Sexual Attitudes and Lifestyles (Natsal) are large probability surveys of sexual attitudes and lifestyles in the British population. Findings from the first survey in 1990–91^12^, ^13^ and the second in 1999–2001^14^, ^15^, ^16^, ^17^ have been used extensively to inform sexual and reproductive health policy in Britain (England, Scotland, and Wales).^18^, ^19^, ^20^ Natsal-3 is the first Natsal survey to include questions about sexual violence and the first population-based survey in Britain to explore the issue outside the context of crime. We asked participants about their experience of sex against their will, which we report as non-volitional sex. We present population estimates for the prevalence of attempted and completed non-volitional sex in women and men since the age of 13 years, the circumstances surrounding the most recent occurrence, and the associations between ever having experienced completed non-volitional sex and several sociodemographic, behavioural, and health factors.

## Methods

### Participants and procedures

Between Sept 6, 2010, and Aug 31, 2012, we interviewed women and men aged 16–74 years living in Britain. We interviewed participants using computer-assisted personal interviews, including a computer-assisted self-interview for the more sensitive questions. Details of the methods used are described elsewhere.^21^, ^22^, ^23^, ^24^ An anonymised dataset will be deposited with the UK Data Archive, and the complete questionnaire and technical report will be available on the Natsal website on the day of publication.

We asked women and men about their experience of sex against their will since the age of 13 years, in the computer-assisted self-interview section of the questionnaire, in which heterosexual sex was defined as including “vaginal, oral, or anal” and same-sex sex as including “oral (or, for men only, anal) sex or any other contact involving the genital area”. Only participants who reported having had heterosexual intercourse or sex with someone of the same sex since 13 years of age were routed to these questions. The first question was worded “Has anyone tried to make you have sex with them, against your will?” Participants who responded “yes” were defined as having experienced “attempted non-volitional sex”, and were then asked “Has anyone actually made you have sex with them, against your will?”, which was used to define the experience of “completed non-volitional sex”. Participants reporting completed non-volitional sex were asked their age at the most recent occurrence and the nature of their relationship with the person responsible (someone you were, or had been, in a relationship with [which we refer to as a current or former intimate partner]; someone known to you as a family member or friend; someone known to you but not as a family member or friend; someone you didn't know; and other). We also asked whether they had told anyone about the experience, and if they had reported it to the police. Immediately after the computer-assisted self-interview section was complete, and before the participant handed the computer back to the interviewer, responses were locked into the computer and could not be accessed by the interviewer. At the end of the interview, we provided all participants with a leaflet detailing organisations offering relevant help and advice.

The Natsal-3 study was approved by the Oxfordshire NHS Research Ethics Committee A (reference: 09/H0604/27). Participants provided oral informed consent for interviews.

### Statistical analysis

We calculated age-specific lifetime population prevalence estimates for reported attempted and completed non-volitional sex and analysed the associations between completed non-volitional sex and a range of factors. Sociodemographic factors included age at interview, family structure at 14 years of age, education, and area-level deprivation (for which we used the Index of Multiple Deprivation, a multidimensional measure combining income, employment, health, education, access to housing and services, crime, and living environment).^25^ Health and behavioural factors included self-reported health status, longstanding illness or disability, treatment for depression or other mental health conditions in the past year, smoking history, frequency of drinking more than six (for women) or eight (for men) units of alcohol per day (ie, binge drinking),^26^ and non-prescription drug use in the past year. Sexual health factors included age at first heterosexual intercourse, ever having a same-sex experience involving genital contact, lifetime number of opposite-sex or same-sex sexual partners, ever having been diagnosed with a sexually transmitted infection, low sexual function (measured using the 17-item Natsal-SF, which includes components on problems with sexual response, sexual function in the relationship context, and self-appraisal of sex life^27^) and, for women, pregnancy outcome before 18 years of age and number of abortions ever.

We did all analyses with the survey commands in Stata (version 12.1), which incorporated the weighting, clustering, and stratification of the Natsal-3 dataset. We used logistic regression to estimate age-adjusted odds ratios to analyse factors associated with the occurrence of completed non-volitional sex in women and men.

### Role of the funding source

The sponsors of the study had no role in study design, data collection, data analysis, data interpretation, or writing of the report. The corresponding author had full access to all the data and had final responsibility for the decision to submit for publication.

## Results

We interviewed 15 162 people (8869 women [median age at interview 43 years] and 6293 men [median age at interview 42 years]). The response rate was 57·7% and the cooperation rate (measured as the number of participants interviewed divided by the number of eligible addresses contacted) was 65·8%. 14 283 participants (8409 women and 5874 men) were routed to the computer-assisted self-interview section of the questionnaire in which they were asked the questions about their experience of non-volitional sex. Of those individuals, 1·7% of women and 1·3% of men reported that they did not know whether this had happened to them, and 2·6% of women and 2·9% of men did not answer the question. We excluded these participants from the analysis. Compared with responders, a higher proportion of item non-responders were of lower educational level, were in the highest quintile of deprivation, and were older (≥55 years for men and ≥65 years for women; data not shown).

Attempted non-volitional sex was reported by 19·4% (95% CI 18·4–20·4) of all women (table 1) and 4·7% (4·1–5·4) of all men (table 2). Half of women (50·5%) and almost a third of men (29·8%) who reported attempted non-volitional sex went on to report completed non-volitional sex, such that completed non-volitional sex was reported by 9·8% (95% CI 9·0–10·5) of women and 1·4% (1·1–1·7) of men. The mean and median age (interdecile range) at the last occurrence of completed non-volitional sex was 20·6 and 18 years (14–32) for women, and 19·2 and 16 years (13–30) for men. The mean and median numbers of years (interdecile range) since the last occurrence were 22·5 and 22 years (5–40) for women, and 23·2 and 22 years (5–48) for men.

**Table 1:** Population prevalence in women of attempted and completed non-volitional sex, by demographic, health, and behavioural factors

		**Attempted non-volitional sex, % (95% CI)**	**Completed non-volitional sex, % (95% CI)**	Age-adjusted odds ratio*(95% CI)	**p value**	**Denominators (unweighted, weighted)**†
All female participants		19·4% (18·4–20·4)	9·8% (9·0–10·5)	..	..	8511, 7332
Age group at interview (years)					<0·0001	
	16–24	16·4% (14·7–18·3)	6·9% (5·8–8·1)	1·00	..	2078, 1172
	25–34	19·1% (17·4–20·9)	9·7% (8·5–11·2)	1·46 (1·16–1·85)	..	2382, 1320
	35–44	21·7% (19·3–24·4)	12·5% (10·5–14·7)	1·93 (1·48–2·52)	..	1171, 1406
	45–54	22·6% (20·0–25·4)	12·2% (10·3–14·3)	1·87 (1·44–2·44)	..	1079, 1387
	55–64	19·8% (17·3–22·5)	10·2% (8·4–12·4)	1·54 (1·16–2·04)	..	987, 1179
	65–74	14·5% (12·0–17·4)	4·9% (3·6–6·7)	0·70 (0·49–1·00)	..	814, 867
Family structure‡					<0·0001	
	Natural or adoptive parents	17·8% (16·7–18·9)	8·5% (7·7–9·3)	1·00	..	6383, 5795
	One natural parent and one step–parent	25·9% (22·3–29·8)	14·5% (11·6–18·0)	1·85 (1·41–2·43)	..	764, 569
	Single parent	24·2% (21·2–27·4)	12·9% (10·5–15·7)	1·62 (1·25–2·09)	..	1132, 776
	In care	45·3% (32·6–58·8)	36·6% (24·7–50·4)	6·22 (3·52–11·00)	..	78, 60
	Other	21·0% (14·6–29·4)	14·3% (8·8–22·3)	1·79 (1·03–3·11)	..	152, 129
Index of Multiple Deprivation§ (quintiles)					0·0019	
	1 (least deprived)	17·9% (15·8–20·2)	7·7% (6·3–9·2)	1·00	..	1567, 1484
	2	18·7% (16·6–21·1)	8·2% (6·8–10·0)	1·09 (0·81–1·45)	..	1647, 1505
	3	22·0% (19·7–24·4)	11·6% (9·8–13·6)	1·59 (1·20–2·10)	..	1681, 1447
	4	19·9% (17·8–22·1)	11·1% (9·5–13·0)	1·52 (1·16–1·99)	..	1776, 1471
	5 (most deprived)	18·5% (16·6–20·7)	10·3% (8·8–12·0)	1·40 (1·07–1·83)	..	1840, 1425
Education at age ≥17 years¶					0·0111	
	No academic qualifications	14·5% (12·7–16·5)	7·9% (6·5–9·4)	1·00	..	1450, 1414
	Academic qualifications typically gained at age 16 years	20·2% (18·5–22·0)	11·0% (9·7–12·5)	1·46 (1·14–1·87)	..	2759, 2430
	Studying for/attained further academic qualifications	21·4% (19·9–23·0)	9·8% (8·7–11·0)	1·27 (0·98–1·66)	..	3841, 3167
Self-reported health status					<0·0001	
	Good/very good	17·9% (16·9–19·0)	8·5% (7·7–9·3)	1·00	..	7003, 5957
	Fair	24·8% (22·0–27·8)	14·0% (11·9–16·3)	1·82 (1·46–2·25)	..	1149, 1033
	Bad/very bad	29·3% (24·2–34·9)	19·9% (15·6–25·0)	2·83 (2·05–3·91)	..	359, 342
Longstanding illness or disability					<0·0001	
	No	17·1% (15·9–18·2)	7·7% (6·9–8·6)	1·00	..	5881, 4879
	Yes	24·1% (22·3–25·9)	13·8% (12·4–15·4)	2·06 (1·71–2·47)	..	2629, 2453
Treatment for depression in the past year‖					<0·0001	
	Not mentioned	17·4% (16·4–18·4)	8·3% (7·5–9·0)	1·00	..	7376, 6414
	Mentioned	33·4% (30·3–36·6)	20·2% (17·7–23·0)	2·82 (2·33–3·41)	..	1133, 916
Treatment for other mental health condition in the past year**					<0·0001	
	Not mentioned	18·8% (17·8–19·8)	9·3% (8·6–10·1)	1·00	..	8293, 7174
	Mentioned	47·1% (39·5–54·9)	31·0% (24·3–38·7)	4·42 (3·12–6·25)	..	216, 156
Smoking history					<0·0001	
	Never	15·4% (14·2–16·7)	6·4% (5·6–7·3)	1·00	..	4422, 3915
	Ex-smoker	23·7% (21·6–26·0)	13·2% (11·5–15·1)	2·24 (1·81–2·78)	..	1796, 1707
	Present	24·3% (22·2–26·5)	14·0% (12·4–15·8)	2·36 (1·93–2·88)	..	2293, 1710
Frequency of binge drinking††					<0·0001	
	Never/rarely	18·2% (17·0–19·4)	9·0% (8·1–9·9)	1·00	..	5475, 4907
	Monthly	20·4% (17·8–23·2)	8·8% (7·1–10·9)	1·00 (0·76–1·31)	..	1134, 857
	At least weekly	25·3% (22·1–28·8)	15·6% (12·9–18·6)	1·89 (1·48–2·42)	..	942, 774
Non-prescription drug use in the past year					<0·0001	
	No	18·5% (17·5–19·5)	9·3% (8·5–10·1)	1·00	..	7572, 6727
	Cannabis only	37·0% (31·7–42·6)	20·6% (16·2–25·7)	2·65 (1·92–3·66)	..	439, 288
	Any hard drug	32·6% (27·0–38·7)	15·2% (10·8–21·1)	1·85 (1·22–2·81)	..	332, 204
First heterosexual intercourse before age 16 years					<0·0001	
	No	16·9% (15·9–18·0)	7·4% (6·7–8·2)	1·00	..	6588, 5986
	Yes	31·5% (29·1–34·1)	20·9% (18·8–23·2)	3·55 (2·96–4·25)	..	1832, 1267
Ever had same-sex experience‡‡					<0·0001	
	No	17·9% (17·0–19·0)	8·6% (7·9–9·4)	1·00	..	7912, 6877
	Yes	41·3% (36·7–46·1)	27·5% (23·4–32·0)	4·10 (3·23–5·21)	..	599, 455
Number of sexual partners (lifetime)§§					<0·0001	
	1	8·3% (6·9–10·0)	1·9% (1·3–2·8)	1·00	..	1586, 1598
	2	13·8% (11·4–16·6)	5·0% (3·5–7·1)	2·79 (1·57–4·93)	..	878, 803
	3–4	15·3% (13·4–17·4)	7·6% (6·3–9·2)	4·41 (2·77–7·03)	..	1525, 1353
	5–9	24·7% (22·5–27·0)	12·3% (10·7–14·1)	7·69 (4·89–12·09)	..	2003, 1687
	≥10	35·7% (33·1–38·3)	21·2% (19·0–23·6)	14·98 (9·55–23·52)	..	1918, 1477
Number of abortions					<0·0001	
	0	17·3% (16·3–18·3)	8·2% (7·5–9·0)	1·00	..	7317, 6332
	1	32·5% (29·0–36·3)	18·0% (15·1–21·2)	2·44 (1·94–3·06)	..	862, 727
	≥2	37·9% (31·4–44·8)	27·3% (21·6–33·8)	4·18 (3·03–5·77)	..	285, 226
First pregnancy outcome before age 18 years¶¶					<0·0001	
	No	18·7% (17·6–19·7)	8·8% (8·1–9·6)	1·00	..	7261, 6521
	Yes	32·6% (28·6–36·8)	23·9% (20·3–27·9)	3·23 (2·57–4·07)	..	711, 521
STI diagnosis ever (excluding thrush)					<0·0001	
	No	16·9% (15·9–17·9)	8·2% (7·5–9·0)	1·00	..	7084, 6246
	Yes	34·5% (31·6–37·4)	18·6% (16·3–21·2)	2·60 (2·15–3·13)	..	1339, 1007
Low sexual function‖‖					<0·0001	
	No	17·8% (16·6–19·0)	8·6% (7·7–9·5)	1·00	..	5378, 4571
	Yes	31·4% (28·4–34·5)	16·8% (14·6–19·4)	2·18 (1·77–2·68)	..	1201, 1135

STI=sexually transmitted infection.

* Odds ratio for a woman's risk of experiencing completed non-volitional sex (relative to not), age-adjusted, with the exception of the age group variable.

† Unweighted, weighted denominators (all participants).

‡ Living circumstances when the participant was 14 years old.

§ Index of Multiple Deprivation (IMD) is a multidimensional measure of area (neighbourhood)-level deprivation based on the participant's postcode. IMD scores for England, Scotland, and Wales were adjusted before being combined and assigned to quintiles, using a method by Payne and Abel.^25^

¶ Denominator excludes women aged 16 years at interview.

‖ Received treatment from a health professional for depression, in the year before interview.

** Received treatment from a health professional for a mental health condition other than depression, in the year before interview.

†† More than six units of alcohol on one occasion.^26^

‡‡ Involving genital contact.

§§ Total number of same-sex partners, opposite-sex partners, or both, excluding those with no partners.

¶¶ Denominator excludes women aged 16–17 years at interview; pregnancy outcome includes livebirth, stillbirth, abortion, or miscarriage.

‖‖ Score uses derived Natsal-3 sexual function measure,^27^ excluding those without a valid score. p values were calculated using the Wald test.

**Table 2:** Population prevalence in men of attempted and completed non-volitional sex, by demographic, health, and behavioural factors

		**Attempted non-volitional sex, % (95% CI)**	**Completed non-volitional sex, % (95% CI)**	Age-adjusted odds ratio*(95% CI)	**p value**	**Denominators (unweighted, weighted)**†
All male participants		4·7% (4·1–5·4)	1·4% (1·1–1·7)	..	..	6049, 7196
Age group at interview (years)					0·0728	
	16–24	3·7% (2·8–4·9)	0·8% (0·5–1·4)	1·00	..	1688, 1208
	25–34	4·4% (3·4–5·7)	1·7% (1·1–2·7)	2·07 (1·03–4·16)	..	1474, 1328
	35–44	4·2% (3·0–5·9)	1·4% (0·8–2·4)	1·67 (0·78–3·60)	..	788, 1394
	45–54	5·9% (4·3–8·0)	1·8% (1·0–3·1)	2·11 (0·95–4·67)	..	764, 1360
	55–64	5·8% (4·2–8·1)	1·6% (0·9–3·0)	1·95 (0·89–4·28)	..	725, 1120
	65–74	4·1% (2·7–6·1)	0·4% (0·1–1·3)	0·47 (0·13–1·69)	..	610, 786
Family structure‡					0·0002	
	Natural or adoptive parents	4·3% (3·6–5·0)	1·1% (0·8–1·5)	1·00	..	4697, 5888
	One natural parent and one step-parent	5·4% (3·5–8·3)	1·2% (0·4–3·1)	1·04 (0·36–2·98)	..	460, 454
	Single parent	7·2% (5·2–9·9)	2·6% (1·6–4·4)	2·39 (1·27–4·51)	..	739, 698
	In care	9·9% (3·0–27·7)	9·9% (3·0–27·7)	9·64 (2·77–33·58)	..	34, 30
	Other	9·2% (4·8–16·9)	3·8% (1·5–9·3)	3·44 (1·25–9·43)	..	119, 125
Index of Multiple Deprivation§ (quintiles)					0·1024	
	1 (least deprived)	3·3% (2·3–4·8)	0·5% (0·2–1·2)	1·00	..	1187, 1488
	2	5·0% (3·8–6·6)	1·5% (0·9–2·6)	3·05 (1·10–8·44)	..	1206, 1532
	3	4·8% (3·6–6·4)	1·7% (1·1–2·8)	3·40 (1·25–9·21)	..	1172, 1398
	4	4·7% (3·6–6·2)	1·3% (0·8–2·1)	2·55 (0·94–6·90)	..	1205, 1426
	5 (most deprived)	5·9% (4·4–7·7)	1·8% (1·2–2·7)	3·54 (1·40–8·97)	..	1279, 1351
Education at age ≥17 years¶					0·2731	
	No academic qualifications	3·4% (2·4–4·7)	0·9% (0·4–1·7)	1·00	..	1056, 1372
	Academic qualifications typically gained at age 16 years	3·9% (3·0–5·0)	1·3% (0·9–2·0)	1·54 (0·64–3·66)	..	1873, 2262
	Studying for/attained further academic qualifications	6·0% (5·1–7·1)	1·7% (1·2–2·3)	1·97 (0·83–4·63)	..	2785, 3284
Self-reported health status					0·0183	
	Good/very good	4·4% (3·8–5·1)	1·2% (0·9–1·5)	1·00	..	4971, 5868
	Fair	6·4% (4·6–8·8)	2·5% (1·6–4·0)	2·28 (1·28–4·04)	..	838, 1037
	Bad/very bad	5·3% (3·1–9·0)	1·2% (0·5–3·0)	1·13 (0·41–3·09)	..	238, 287
Longstanding illness or disability					0·0046	
	No	4·0% (3·4–4·8)	1·1% (0·8–1·5)	1·00	..	4285, 4911
	Yes	6·2% (5·1–7·5)	2·0% (1·4–2·8)	2·02 (1·24–3·28)	..	1763, 2284
Treatment for depression in the past year‖					<0·0001	
	Not mentioned	4·3% (3·7–5·0)	1·1% (0·8–1·5)	1·00	..	5635, 6753
	Mentioned	11·0% (8·0–14·8)	5·1% (3·2–8·0)	4·80 (2·75–8·37)	..	413, 442
Treatment for other mental health condition in the past year**					0·0225	
	Not mentioned	4·6% (4·1–5·3)	1·3% (1·0–1·7)	1·00	..	5893, 7043
	Mentioned	8·6% (5·1–14·2)	3·4% (1·6–7·3)	2·67 (1·15–6·20)	..	155, 152
Smoking history					0·0001	
	Never	3·7% (3·0–4·6)	0·6% (0·3–1·0)	1·00	..	2935, 3403
	Ex-smoker	5·1% (4·0–6·5)	1·5% (1·0–2·3)	2·63 (1·23–5·64)	..	1371, 1906
	Present	6·2% (4·9–7·6)	2·6% (1·8–3·6)	4·44 (2·27–8·68)	..	1743, 1886
Frequency of binge drinking††					0·4245	
	Never/rarely	4·7% (3·9–5·6)	1·5% (1·1–2·0)	1·00	..	3362, 4195
	Monthly	5·3% (3·8–7·3)	1·5% (0·8–2·7)	1·02 (0·52–2·02)	..	1020, 1127
	At least weekly	4·1% (3·1–5·5)	0·9% (0·5–1·7)	0·64 (0·32–1·28)	..	1242, 1403
Non-prescription drug use in the past year					0·0005	
	No	4·3% (3·7–5·1)	1·1% (0·8–1·5)	1·00	..	4729, 5985
	Cannabis only	4·6% (3·1–6·8)	2·1% (1·1–3·8)	2·10 (0·97–4·56)	..	654, 612
	Any hard drug	10·7% (8·0–14·2)	3·7% (2·2–6·1)	3·78 (1·92–7·43)	..	510, 471
First heterosexual intercourse before age 16 years					0·0102	
	No	3·7% (3·1–4·3)	1·1% (0·8–1·5)	1·00	..	4408, 5375
	Yes	7·6% (6·1–9·3)	2·1% (1·5–3·0)	1·91 (1·17–3·13)	..	1576, 1738
Ever had same-sex experience‡‡					<0·0001	
	No	3·8% (3·3–4·4)	0·8% (0·6–1·2)	1·00	..	5700, 6795
	Yes	20·0% (15·7–25·2)	10·1% (7·1–14·3)	13·31 (7·93–22·35)	..	349, 400
Number of sexual partners (lifetime)§§					0·0153	
	1	1·7% (0·9–3·0)	0·4% (0·1–1·9)	1·00	..	757, 937
	2	2·3% (1·3–4·0)	0·3% (0·1–1·2)	0·70 (0·09–5·59)	..	477, 579
	3–4	3·2% (2·0–5·0)	0·9% (0·3–2·2)	2·13 (0·35–12·86)	..	852, 1043
	5–9	4·3% (3·3–5·6)	1·9% (1·2–2·9)	4·71 (0·94–23·72)	..	1378, 1707
	≥10	7·7% (6·4–9·1)	2·0% (1·4–2·7)	4·99 (1·04–23·96)	..	2049, 2477
STI diagnosis ever (excluding thrush)					0·0001	
	No	3·7% (3·1–4·3)	1·1% (0·8–1·4)	1·00	..	5245, 6217
	Yes	11·1% (8·7–14·0)	3·2% (2·0–4·9)	2·97 (1·74–5·07)	..	733, 893
Low sexual function¶¶					0·0206	
	No	4·1% (3·4–4·9)	1·2% (0·8–1·6)	1·00	..	3900, 4774
	Yes	7·3% (5·6–9·4)	2·2% (1·4–3·5)	1·97 (1·11–3·50)	..	912, 1175

STI=sexually transmitted infection.

* Odds ratio for a man's risk of experiencing completed non-volitional sex (relative to not), age-adjusted, with the exception of the age group variable.

† Unweighted, weighted denominators (all participants).

‡ Living circumstances when participant was 14 years old. §Index of Multiple Deprivation (IMD) is a multidimensional measure of area (neighbourhood)-level deprivation based on the participant's postcode. IMD scores for England, Scotland, and Wales were adjusted before being combined and assigned to quintiles, using a method by Payne and Abel.^25^

¶ Denominator excludes men aged 16 years at interview.

‖ Received treatment from a health professional for depression, in the year before interview.

** Received treatment from a health professional for a mental health condition other than depression, in the year before interview.

†† More than eight units of alcohol on one occasion.^26^

‡‡ Involving genital contact.

§§ Total number of same-sex partners, opposite-sex partners, or both, excluding those with no partners.

¶¶ Score uses derived Natsal-3 sexual function measure,^27^ excluding those without a valid score. p values were calculated using the Wald test.

The prevalence of reported experience of attempted and completed non-volitional sex varied by several sociodemographic characteristics in both women and men (Table 1, Table 2). In women, ever having experienced either event was reported less often by the youngest (aged 16–24 years) and oldest (aged 65–74 years) participants. In men, the reported prevalence was similar across all age groups. Notable differences occurred in the prevalence of attempted and completed non-volitional sex by family structure. Non-volitional sex was reported more frequently by women and men who grew up in single parent or “other” households or in care, and by women who lived with one natural parent and one step-parent. The strong association seen between experience of completed non-volitional sex and growing up in care should be interpreted with caution in view of the small number of participants for whom this was the case. In women, completed non-volitional sex was associated with currently (at the time of interview) living in more deprived areas and, conversely, with higher educational attainment; the associations for both these variables in men were in the same direction but were not statistically significant (table 2).

Reporting of attempted and completed non-volitional sex was higher in women who rated their overall health as bad or very bad, or fair (table 1) and in men who rated their health as fair (table 2) than in those rating it as good or very good. Both experiences were also more common in women and men reporting a longstanding illness or disability, and among those who had received treatment for either depression or another mental health condition in the year before interview, compared with those who had not.

In women and men reporting past or present smoking, or use of non-prescription drugs in the year before interview, and in women who reported binge drinking at least weekly, experience of attempted and completed non-volitional sex was higher than in those not reporting these behaviours (Table 1, Table 2).

Attempted and completed non-volitional sex also varied by several sexual behaviour variables and by a range of sexual health indicators (Table 1, Table 2). Prevalences of both attempted and completed non-volitional sex were higher in women and men reporting first heterosexual intercourse before age 16 years, same-sex experience, a higher number of lifetime sexual partners, ever being diagnosed with a sexually transmitted infection, low sexual function, and, in women, those reporting abortion and pregnancy outcome before 18 years of age.

We recorded strong associations after adjusting for age (age-adjusted odds ratio) with completed non-volitional sex and all these behavioural and health factors (Table 1, Table 2), with the exception of binge drinking in men. All associations, including those with the sociodemographic characteristics described previously, were sustained for women after further adjustment for family structure, area-level deprivation, and education (appendix). We could not make the same adjustments for men because of the small number of male participants reporting completed non-volitional sex.

**Figure 1:**
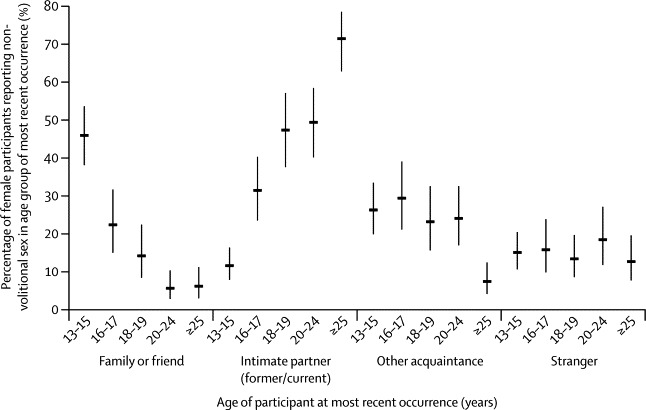
Perpetrator at most recent occurrence of completed non-volitional sex by age group of most recent occurrence (women only) Vertical lines through plotted points are 95% CIs. The denominator is the weighted number of women reporting completed non-volitional sex ever.

In most instances of completed non-volitional sex, the perpetrator was known to the participant, either as a current or former intimate partner (40·6% women, 22·9% men), a family member or friend (20·4% women, 30·2% men), or known to them but not as a family member or friend (20·8% women, 29·7% men). In only a few instances was the person responsible a stranger (14·8% women, 15·3% men). The nature of the relationship with the perpetrator varied with the age at last occurrence (figure 1), except when that person was a stranger. The proportion of cases in which a family member or friend was identified as the perpetrator decreased with increasing age, from 45·3% in women aged 13–15 years to 5·8% of those aged 25 years and older at the most recent occurrence (figure 1). Where intimate partners were the perpetrators, the opposite pattern was evident: 11·4% of women aged 13–15 years at the most recent occurrence identified the person responsible as someone with whom they were or had been in a relationship, which increased to 71·5% of those aged 25 years and older (figure 1). The corresponding data for men are not shown because of the small numbers.

**Figure 2:**
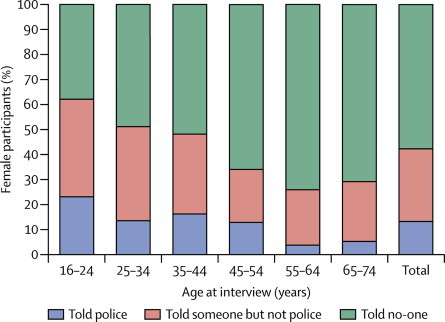
Most recent occurrence of completed non-volitional sex (women only) The denominator is the weighted number of women reporting completed non-volitional sex ever.

**Figure 3:**
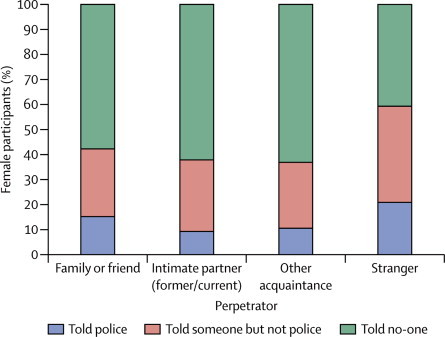
Most recent occurrence of completed non-volitional sex by perpetrator (women only) The denominator is the weighted number of women reporting completed non-volitional sex ever.

Of the participants who reported completed non-volitional sex, fewer than half told someone about the event, although women were more likely to have done so than were men (42·2% of women *vs* 32·6% of men). Women were also more likely than men to have reported the event to the police (12·9% of women *vs* 8·0% of men). The proportion of women who either told someone or reported the event to the police varied by age at interview (figure 2) and by perpetrator (figure 3). The proportion of women reporting to the police increased with younger age at interview, and was higher when the perpetrator was a stranger (20·9% of women reported the act when it was committed by a stranger compared with 9·4% when it was committed by a current or former intimate partner). Again, the corresponding figures for men are not shown because of the small numbers involved.

## Discussion

Our data show that one in five women and one in 20 men in Britain report experiencing attempted non-volitional sex, and one in ten women and one in 71 men report experiencing completed non-volitional sex since age 13 years. We have used the term “non-volitional sex” as the most literal translation of the question asked. Irrespective of the degree of coercion or force used, it represents a violation of sexual autonomy and is therefore a form of sexual violence. Worldwide, prevalence estimates of sexual violence vary substantially.^1^ However, direct comparisons are difficult to make because of differences in the framing of surveys, the measures used, the methods employed, and the population under study.^3^, ^28^ The American National Intimate Partner and Sexual Violence Survey and the French Context of Sexuality in France survey, which, like Natsal-3 are national probability sample surveys, showed similar levels of reporting.^29^, ^30^

In Britain, the only existing population data come from the Crime Survey for England and Wales^31^ in which the prevalence of ever experiencing completed “rape”—3·8% in women and 0·2% in men—is lower than our estimates for non-volitional sex. If we restrict Natsal-3 data to participants aged 16–59 years and to occurrences after the age of 16 years (as per the Crime Survey for England and Wales), our estimates are still higher than those of the Crime Survey, at 7·5% for women and 0·8% for men. The difference is probably due to a combination of variations in methods, wording of questions, and context. The questions in the Crime Survey for England and Wales are designed to specifically measure rape as legally defined, as opposed to the broader definition of non-volitional sex used here. However, asking about experiences in a crime survey could result in under-reporting because participants might only include events that they perceive as illegal^32^ and, as noted in the introduction, many people who have experienced what would be legally defined as rape do not acknowledge it as such.^10^ Where our data do concur with the Crime Survey for England and Wales^31^ is in the nature of the relationship with the perpetrator, who is most often someone known to the individual, and we also show similar low levels of reporting to the police (panel).^31^

Panel **Research in context**

**Systematic review**

The first global systematic review of the prevalence and health effects of violence against women estimated that 35·6% of women have experienced physical violence, sexual violence, or both, at some point in their lives, with regional estimates ranging from 27·2% in Europe to 45·6% in Africa, and concluded that the experience is strongly associated with poorer physical, sexual and reproductive, and mental health outcomes.^1^ Less is known about the prevalence and associated outcomes in men.^8^ So far, information about sexual violence in England and Wales has relied on data from the annual crime survey.^31^ Crime surveys are limited in scope with regard to the exploration of potential factors associated with the experience, and measurement of sexual violence in the context of crime is thought to underestimate prevalence.^29^, ^32^ Natsal-3 is the first of the Natsal surveys to ask questions about sexual violence. We asked women and men about their experience of sex against their will, which, in the most literal interpretation of the question, we report as non-volitional sex.

**Interpretation**

Our estimates for the prevalence of non-volitional sex in women and men are higher than those for the more narrowly defined experience of “rape” found in the 2011/12 Crime Survey for England and Wales^31^ but are similar to estimates from non-crime population surveys in other high-income countries.^29^, ^30^ Our findings concur with those of the Crime Survey for England and Wales in terms of the low level of reporting to the police and in the nature of the relationship with the perpetrator, who is most often someone known to the individual. We recorded strong associations between experience of non-volitional sex and health and behavioural factors in both women and men.

As reported elsewhere,^1^, ^29^, ^30^ we showed non-volitional sex to be mainly an experience of young age, with a median age at the most recent occurrence of 18 years in women and 16 years in men. Two groups known to be vulnerable to sexual victimisation—corroborated in our data—are men who have had sex with men^29^, ^33^, ^34^, ^35^ and people who grow up in care.^36^ However, the latter finding should be interpreted cautiously because of the small number of participants to whom this applies. Moreover, we do not know whether participants encountered abuse while in care; men and women could have been placed in care because of sexual abuse in the home or they might have been more vulnerable to sex against their will in their other relationships.^37^

Our findings show strong and consistent associations between experience of completed non-volitional sex and poor mental and physical health status and potentially harmful health behaviours. Since reporting of these health factors was close to the time of interview (or the preceding 12 months), we know them to have been experienced subsequent to the occurrence of non-volitional sex, but they might also have occurred before the event, and therefore a direction of effect cannot be established. The association between IPV and mental health, especially in women, is now well established in the scientific literature.^1^, ^5^, ^38^, ^39^ However, evidence also suggests that people with mental health disorders are more vulnerable to sexual assault than are those without such disorders.^40^, ^41^ Longitudinal studies^42^ indicate that the association between IPV and depression is bidirectional, although sexual violence has not been studied in isolation from other forms of violence. Sexual violence and depression also share common risk factors for which we were not able to adjust, especially childhood exposure to abuse and socioeconomic disadvantage;^42^ we did not ask about the former, and the information that we have about the latter refers to current conditions and not those at the time of the event. Furthermore, related experiences could have cumulative effects. Disability, for example, has been identified as a risk factor for sexual violence^40^, ^43^, ^44^ and victims of sexual violence with a disability—especially those with pre-existing mental illness—are more likely to experience mental health problems after violent incidents than are those without, which compounds the harm caused.^40^ Additionally, research suggests that, in the context of IPV, few women are victims of solely sexual abuse.^3^, ^38^, ^45^

Similarly, we cannot establish the direction of effect with respect to the notable associations in our report between experience of non-volitional sex and a range of indicators of sexual behaviour and sexual health, including first heterosexual intercourse before age 16 years, number of sexual partners, sexually transmitted infection diagnosis, and low sexual function in both sexes, and with abortion and first pregnancy outcome before 18 years of age in women. Many of these associations could be the direct result of non-volitional sex, or they might be linked indirectly through a common cause, such as reduced sexual agency, increased risk behaviours, or both.^1^

The strength of this study lies in the size and nature of the sample, which was selected randomly and is nationally representative, and in its methods, in particular the use of computer-assisted self-interview^46^ to maximise reporting and confidentiality of responses. Arguably, a further strength relates to the fact that the questions about experience of non-volitional sex were asked in the context of a sexual behaviour survey, as opposed to a crime or general health survey.

Several limitations, however, should be considered. First, our data rely on answers to a single, broadly worded question, and its interpretation by participants might have differed by age and sex. Second, the question as worded covers a wide range of experiences that we cannot distinguish between; we did not ask about frequency, severity, the number of perpetrators or their sex, or other details such as the involvement of drugs or alcohol (although it should be noted that the law governing rape in the UK does not require the victim to have physically resisted and covers circumstances in which the victim does not have the capacity to consent^47^). Third, the data are susceptible to biases associated with both response and reporting. In terms of response bias, the numbers that we report could be under-estimates of non-volitional sex because those most at risk might be under-represented; vulnerable groups such as the homeless and people living in institutions are excluded because of the sampling strategy^22^ and those in abusive relationships at the time of interview might have been less likely to participate. Although people not included in the sampling frame might be at increased risk of sexual violence, they also account for a small proportion of the population;^48^ as such, we believe the effect on estimates at the population level is likely to be negligible. Within the survey, only participants who reported that they had had heterosexual intercourse or sex with someone of the same sex since 13 years of age were routed to the computer-assisted self-interview, in which we asked the questions about non-volitional sex. We have assumed that participants not routed into the computer-assisted self-interview have not experienced sex against their will; however, some participants who did not report sex might actually have experienced attempted non-volitional sex but did not have the opportunity to report it. Moreover, participants whose only sexual experience was forced might have not reported it and so would not have progressed to the questions. Additionally, in view of the sensitive nature of the topic, participants might have chosen not to disclose the experience; this non-disclosure could also have been related to older age at interview. It is also possible that people who report poor health are more likely to recall or report experience of negative events,^5^ although research suggests that disclosure is more likely to be affected by issues with methods than by the personal characteristics of the participants.^11^, ^49^

Several important implications for research, policy, and practice stem from these findings. In terms of research, longitudinal data are needed to establish the direction of effects, and qualitative data are needed to gain a better understanding of the associations seen. We also know less about the perpetrators and about effective means of prevention. In terms of policy and practice, first, non-volitional sex is mainly an experience of young age and research suggests that those who suffer sexual abuse early in life are more likely to be revictimised,^30^ which emphasises that early intervention is essential. The UK Government plans to promote the teaching of “sexual consent and the importance of healthy relationships in schools”;^50^ however non-biological aspects of sex and relationship education are currently not compulsory in the curriculum, and as such implementation might be hindered. Second, these data suggest that some people are more vulnerable to sex against their will than are others, which supports the case for targeted intervention. Third, although some evidence in these data suggests that the younger participants in the survey were more likely than older participants to speak to someone about the occurrence of non-volitional sex and to report it to the police, much silence remains around the issue. We need to raise awareness of the issue and to de-stigmatise reporting.

The clustering of adverse sexual health risks argues for vigilance in a public health context for links between risk factors, and for the adoption of a holistic view of sexual health in both preventive and therapeutic interventions. Furthermore, the wide range of health and sexual health-related variables associated with non-volitional sex calls for integrated services for victims. Health professionals should be cognisant and ask specifically about experience of sexual violence when people present for other issues, especially since the effects can be long lasting. Finally, our data argue for greater efforts to counter myths and misconceptions, such as the stereotype of the perpetrator as a “stranger in the bushes”. The strategies needed to achieve these broader goals go beyond the realms of public health and extend to all areas of society.

## Supplementary Material

Supplementary appendix

Supplementary video Results of over 45,000 respondents, recording the dramatic changes in our sexual lifestyles over the past 60 years.
