# Supplementary material for: Lifetime prevalence, associated factors, and circumstances of non-volitional sex in women and men in Britain: findings from the third National Survey of Sexual Attitudes and Lifestyles (Natsal-3)
Source: Lancet. 2013 Nov 30;382(9907):1845–55. doi: 10.1016/S0140-6736(13)62300-4 (PMC3898964; doi:10.1016/S0140-6736(13)62300-4)
Supplement: Supplementary appendix [file mmc1.pdf]

THE LANCET

Supplementary appendix

This appendix formed part of the original submission and has been peer reviewed. We post it as supplied by the authors.

Supplement to: Macdowall W, Gibson LJ, Tanton C, et al. Lifetime prevalence, associated factors, and circumstances of non-volitional sex in women and men in Britain: findings from the third National Survey of Sexual Attitudes and Lifestyles (Natsal-3). *Lancet* 2013; published online Nov 26. http://dx.doi.org/10.1016/S0140-6736(13)62300-4.

Appendix 1: Logistic regression of completed non-volitional sex, by demographic, health and behavioural factors, women

	aAOR[1]	95% CI[2]	p-value	AOR[3]	95% CI[2]	p-value
**Age group at interview**			<0·0001			<0·0001
16-24	1·00			1.00		
25-34	1·46	(1·16-1·85)		1.40	(1·09-1·79)	
35-44	1·93	(1·48-2·52)		2.05	(1·54-2·71)	
45-54	1·87	(1·44-2·44)		2.09	(1·57-2·79)	
55-64	1·54	(1·16-2·04)		1.84	(1·32-2·56)	
65-74	0·70	(0·49-1·00)		0.92	(0·61-1·37)	
**Family circumstances**[4]			<0·0001			<0·0001
with natural/adoptive parents	1·00			1·00		
with one natural/one step	1·85	(1·41-2·43)		1·92	(1·45-2·55)	
with single parent	1·62	(1·25-2·09)		1.62	(1·25-2·11)	
in care	6·22	(3·52-11·00)		7.03	(3·93-12·59)	
other	1·79	(1·03-3·11)		1.64	(0·90-2·99)	
**Index of Multiple Deprivation**[5] **(quintiles)**			0·0019			0·0023
1 (least deprived)	1·00			1·00		
2	1·09	(0·81-1·45)		1·08	(0·81-1·44)	
3	1·59	(1·20-2·10)		1·59	(1·19-2·11)	
4	1·52	(1·16-1·99)		1·54	(1·17-2·04)	
5 (most deprived)	1·40	(1·07-1·83)		1·42	(1·07-1·89)	
**Education at age 17+**[6]			0·0111			0·0004
no academic qualifications	1·00			1·00		
academic qualifications typically gained at age 16	1·46	(1·14-1·87)		1·66	(1·29-2·15)	
studying for/attained further academic qualifications	1·27	(0·98-1·66)		1·62	(1·23-2·15)	
**Self-reported health status**			<0·0001			<0·0001
good/very good	1·00			1·00		
fair	1·82	(1·46-2·25)		1·84	(1·47-2·31)	
bad/very bad	2·83	(2·05-3·91)		2·77	(2·01-3·84)	
**Longstanding illness or disability**			<0·0001			<0·0001
no	1·00			1·00		
yes	2·06	(1·71-2·47)		2·04	(1·69-2·46)	
**Treatment for depression in past year**[7]			<0·0001			<0·0001
not mentioned	1·00			1·00		
mentioned	2·82	(2·33-3·41)		2·64	(2·16-3·22)	
**Treatment for other mental health condition in past year**[8]			<0·0001			<0·0001
not mentioned	1·00			1·00		
mentioned	4·42	(3·12-6·25)		4·14	(2·90-5·91)	
**Smoking history**			<0·0001			<0·0001
never	1·00			1·00		
ex-smoker	2·24	(1·81-2·78)		2·23	(1·78-2·78)	
current	2·36	(1·93-2·88)		2·36	(1·89-2·94)	
**Frequency of binge drinking**[9]						
never/rarely	1·00		<0·0001	1·00		<0·0001
monthly	1·00	(0·76-1·31)		1·01	(0·77-1·33)	
at least weekly	1·89	(1·48-2·42)		1·79	(1·40-2·30)	
**Non-prescription drug use in past year**			<0·0001			<0·0001
no	1·00			1·00		
cannabis only	2·65	(1·92-3·66)		2·77	(2·00-3·84)	
any hard drug	1·85	(1·22-2·81)		1·76	(1·14-2·71)	
**First heterosexual intercourse before age 16**			<0·0001			<0·0001
no	1·00			1·00		
yes	3·55	(2·96-4·25)		3·41	(2·81-4·13)	
**Ever had same sex experience**[10]			<0·0001			<0·0001
no	1·00			1·00		
yes	4·10	(3·23-5·21)		3·68	(2·87-4·70)	
**Number of sexual partners (lifetime)**[11]			<0·0001			<0·0001

	1	1·00			1·00		
	2	2·79	(1·57-4·93)		2·86	(1·58-5·15)	
	3-4	4·41	(2·77-7·03)		4·17	(2·57-6·79)	
	5-9	7·69	(4·89-12·09)		7·26	(4·50-11·70)	
	10+	14·98	(9·55-23·52)		14·33	(8·93-23·01)	
**Number of abortions**				<0·0001			<0·0001
	0	1·00			1·00		
	1	2·44	(1·94-3·06)		2·28	(1·81-2·87)	
	2+	4·18	(3·03-5·77)		4·00	(2·88-5·56)	
**First pregnancy under age 18**[12]				<0·0001			<0·0001
	no	1·00			1·00		
	yes	3·23	(2·57-4·07)		3·07	(2·42-3·91)	
**STI**[13] **diagnosis ever (excluding thrush)**				<0·0001			<0·0001
	no	1·00			1·00		
	yes	2·60	(2·15-3·13)		2·30	(1·90-2·79)	
**Low sexual function**[14]				<0·0001			<0·0001
	no	1·00			1·00		
	yes	2·18	(1·77-2·68)		2·09	(1·69-2·58)	

[1] odds ratio for a woman's risk of experiencing completed non-volitional sex (relative to not), age-adjusted except for 'Age Group'
[2] 95% Confidence Interval
[3] odds ratio for a woman's risk of experiencing completed non-volitional sex (relative to not), adjusted for age, living circumstances, deprivation and education
[4] living circumstances when participant was age 14
[5] a measure of relative deprivation for the UK, divided in quintiles (30)
[6] denominator excludes women aged 16 at interview
[7] received treatment from a health professional for depression, in the year prior to interview
[8] received treatment from a health professional for a mental health condition other than depression, in the year prior to interview
[9] more than 6 units on one occasion (31)
[10] involving genital contact
[11] total number of same and/or opposite sex partners, excluding those with no partners
[12] denominator excludes women aged 16-17 at interview
[13] Sexually Transmitted Infection
[14] score using derived Natsal-3 sexual function measure (32), excluding those without a valid score
